# A machine learning approach to explore predictors of graft detachment following posterior lamellar keratoplasty: a nationwide registry study

**DOI:** 10.1038/s41598-022-22223-y

**Published:** 2022-10-21

**Authors:** M. B. Muijzer, C. M. W. Hoven, L. E. Frank, G. Vink, R. P. L. Wisse, The Netherlands Corneal Transplant Network (NCTN), Marjolijn C. Bartels, Yanny Y. Cheng, Mario R. P. Dhooge, Mor Dickman, Bart T. H. van Dooren, Cathrien A. Eggink, Annette J. M. Geerards, Tom A. van Goor, Ruth Lapid-Gortzak, Chantal M. van Luijk, Ivanka J. van der Meulen, Carla P. Nieuwendaal, Rudy M. M. A. Nuijts, Siamak Nobacht, Abdulkarim Oahalou, Emile C. A. A. van Oosterhout, Lies Remeijer, Jeroen van Rooij, Nathalie T. Y. Santana, Remco Stoutenbeek, Mei L. Tang, Thijs Vaessen, Nienke Visser, Robert H. J. Wijdh, Robert P. L. Wisse

**Affiliations:** 1 Department of Ophthalmology, University Medical Center Utrecht, Heidelberglaan 100, 3584 CX Utrecht, The Netherlands; 2 Department of Methodology and Statistics, Utrecht University, Utrecht, the Netherlands; 3 Deventer Hospital, Deventer, The Netherlands; 4 Leiden University Medical Center, Leiden, The Netherlands; 5 Elkerliek Hospital, Helmond, The Netherlands; 6 University Eye Clinic, Maastricht University Medical Center, Maastricht, The Netherlands; 7 Erasmus Medical Center, Rotterdam, The Netherlands; 8 Amphia Hospital, Breda, The Netherlands; 9 Radboud University Medical Center, Nijmegen, The Netherlands; 10 Eye hospital Rotterdam, Rotterdam, The Netherlands; 11 Amsterdam University Medical Center, Amsterdam, The Netherlands; 12 Gelre Hospitals, Apeldoorn, The Netherlands; 13 University Medical Center Groningen, Amsterdam, The Netherlands

**Keywords:** Predictive markers, Outcomes research

## Abstract

Machine learning can be used to explore the complex multifactorial patterns underlying postsurgical graft detachment after endothelial corneal transplantation surgery and to evaluate the marginal effect of various practice pattern modulations. We included all posterior lamellar keratoplasty procedures recorded in the Dutch Cornea Transplant Registry from 2015 through 2018 and collected the center-specific practice patterns using a questionnaire. All available data regarding the donor, recipient, surgery, and practice pattern, were coded into 91 factors that might be associated with the occurrence of a graft detachment. In this research, we used three machine learning methods; a regularized logistic regression (lasso), classification tree analysis (CTA), and random forest classification (RFC), to select the most predictive subset of variables for graft detachment. A total of 3647 transplants were included in our analysis and the overall prevalence of graft detachment was 9.9%. In an independent test set the area under the curve for the lasso, CTA, and RFC was 0.70, 0.65, and 0.72, respectively. Identified risk factors included: a Descemet membrane endothelial keratoplasty procedure, prior graft failure, and the use of sulfur hexafluoride gas. Factors with a reduced risk included: performing combined procedures, using pre-cut donor tissue, and a pre-operative laser iridotomy. These results can help surgeons to review their practice patterns and generate hypotheses for empirical research regarding the origins of graft detachments.

## Introduction

Posterior lamellar keratoplasty is the current standard treatment to restore visual function in patients with irreversible corneal endothelial cell dysfunction^1^. Two principal treatment modalities are currently used, namely Descemet’s stripping endothelial keratoplasty (DSEK) and Descemet’s membrane endothelial keratoplasty (DMEK)^2^. In recent years, DMEK has increased in popularity due to its potential for faster visual recovery and improved visual outcome compared to DSEK^3,4^. In both DSEK and DMEK, postoperative detachment of the graft is a relatively common complication^5–7^. Detachment can require a secondary surgical intervention, potentially resulting in a less viable graft. The reported prevalence of graft detachment ranges from 2 to 27% for DSEK and 6% to 82% for DMEK^5–7^.

The underlying cause of graft detachment is considered to be multifactorial^8–12^, and a wide range of risk factors have been proposed and/or investigated relating to the type of procedure (DSEK versus DMEK)^5–7^, graft storage and preparation (e.g., pre-cut versus surgeon-cut)^13–16^, and recipient and donor’s characteristics (e.g., prior corneal transplantation, cause of death)^8,11,12,16–18^. Furthermore, various “best practice patterns” have been proposed, resulting in a wide range of surgical tools and techniques that have been adopted when performing posterior lamellar keratoplasty^9,19^. These include the insertion method^8,20,21^, the size of the descemetorhexis^22,23^, combined surgical procedures^12,24–27^, the use of anterior chamber (AC) tamponade (e.g., the agent, volume, pressure, and duration)^9,12,19,24–26,28–33^, and the duration of time spent in the supine position (imposed or recommended) following surgery^9,28^.

In the Netherlands, all centers that perform corneal transplants report their procedures to the Netherlands Organ Transplant Registry (NOTR). These records include extensive follow-up data, including complications, thus providing a unique source of real-world data regarding graft survival, patient characteristics, and donor characteristics. We expanded this dataset using a 35-item questionnaire regarding the preoperative, perioperative, and postoperative procedures performed at each center, as rapidly changing practice patterns in the field of surgery made performing an independent assessment of these practice patterns in a clinical study unfeasible.

Machine learning models can be used to detect complex patterns in large datasets, and these patterns can help researchers identify factors that can predict the risk of postsurgical complications^34,35^. Here, we present the results of our machine learning analysis to identify factors that predict an increase or decrease in the risk of graft detachment following posterior lamellar keratoplasty.

## Results

A total of 3647 posterior lamellar keratoplasties were performed in the Netherlands and recorded in the NOTR registry between January 1, 2015 and December 31, 2018, including 2651 DSEK procedures (73%) and 996 DMEK procedures (27%). The surgeries were performed at sixteen centers throughout the Netherlands. Twelve of these centers submitted their practice patterns (Supplementary Tables S1 and S2), while the other four centers did not respond to the survey. These four centers performed 227 DSEK procedures and 1 DMEK procedure; for these four centers, only the NOTR data were included in the analysis. None of the continuous explanatory variables had missing observations. Sixteen categorical explanatory variables had missing values (mean percentage of missing values: 2.7% SD ± 4.3%; range: 5–20%); these values were recorded as “unknown” and were included for analysis as a new category in the respective variable.

### Donor, recipient and procedure characteristics

The mean (± SD) donor age was 70 ± 9 years, and 61.4% of donors were male. The most common cause of death was diseases of the circulatory system (53.3%), followed by diseases of the respiratory system (16%), other/unknown (20.3%), cancer (8.8%), and trauma (1.5%). The mean interval between death and the transplant procedure was 19 ± 5 days. A complete summary of the donor characteristics is provided in Supplementary Table S3.

The most common indication for surgery was FECD (76.7% of cases). Interestingly, FECD was the indication for performing DMEK in 91.1% of cases, compared to 71.2% in DSEK. The majority of recipients were pseudophakic prior to surgery, with 62.3% having a posterior chamber intraocular lens. In total, 88.8% of recipients had not previously undergone a corneal transplant in the same eye. In 39.5% of cases, the graft was equal in size to the descemetorhexis; the graft was undersized in 26.1% of cases and oversized in 34.4% of cases. In 71.7% of cases, posterior lamellar keratoplasty was not combined with another surgical procedure. The most frequently performed combined surgical procedures were peripheral iridectomy and cataract surgery (12.2% and 8.6% of cases, respectively). A surgical complication was reported in the NOTR in 4.1% of all cases (3.3% of DSEK procedures and 6% of DMEK procedures) and included endothelial damage (0.8% of cases), difficulty unfolding the graft (0.7%), graft rupture or preparation problems (0.4%), iris prolapse (0.4%), and hemorrhage of the AC (0.4%). The recipient and surgery characteristics are summarized in Supplementary Tables S4 and S5, respectively.

### Postoperative complications

The incidence and prevalence of postoperative graft detachment are summarized in Table 1. Overall, the rate of graft detachment was 9.9% (361 out of the 3647 procedures performed over the 4-year study period). During the period from 2015 through 2018, the number of DMEK procedures performed each year increased considerably from 4 to 473, while the number of DSEK procedures performed each year decreased from 743 to 560, reflecting the growing preference for this newer surgical procedure. The prevalence of graft detachment was relatively stable among the patients who underwent DSEK, ranging from 6.2 to 8.4%; in contrast, the prevalence of graft detachment was generally higher among the patients who underwent DMEK, ranging from 11.8 to 14%.

**Table 1:** Summary of graft detachments following DSEK and DMEK procedures performed in the Netherlands from 2015 through 2018.

	DSEK (n = 2651)			DMEK (n = 996)			All procedures (n = 3647)		
	Procedures performed (n)	Detachments, n (%)	Prevalence per center, mean ± SD (%)	Procedures performed (n)	Detachments, n (%)	Prevalence per center, mean ± SD (%)	Procedures performed (n)	Detachments, n (%)	Prevalence per center, mean ± SD (%)
**Year**									
2015–2018	2651	188 (7.1)	8.4 ± 7.2	996	173 (17.4)	13.4 ± 8.2	3647	361 (9.9)	10 ± 6.7
2015	743	46 (6.2)	2.2 ± 2.8	43	6 (14.0)	0.04 ± 0.8	786	52 (6.6)	1.8 ± 2.6
2016	716	45 (6.3)	2.6 ± 3.6	213	54 (25.4)	4.1 ± 3.2	929	99 (10.7)	3.3 ± 3.3
2017	632	50 (7.9)	2.2 ± 1.6	267	57 (21.3)	4.5 ± 2.8	899	107 (11.9)	2.8 ± 1.6
2018	560	47 (8.4)	1.5 ± 1.6	473	56 (11.8)	4.4 ± 5.2	1033	103 (10.0)	2.2 ± 2.2

*DSEK* Descemet’s stripping endothelial keratoplasty, *DMEK* Descemet’s membrane endothelial keratoplasty, *SD* standard deviation.

### Results of the machine learning models

After correction for the DMEK learning curve, a total of 3464 cases were included for machine learning analysis. The discriminatory power of the three machine learning models at predicting graft detachment (based on the AUC) ranged from 0.65 to 0.72 (Table 2). The sensitivity and specificity were similar both within and between models, indicating a similar ability to predict both detachment and non-detachments, and indicating that a considerable amount of variation was not captured by the predictive factors included in the dataset.

**Table 2:** The performance of the three machine learning models at predicting graft detachment in the test dataset.

Prediction model	AUC	Sensitivity^a^	Specificity
LASSO logistic regression	0.70	0.70	0.65
Classification tree analysis	0.65	0.65	0.62
Random forest classifier	0.72	0.68	0.62

*AUC* area under the curve, *LASSO* least absolute shrinkage and selection operator.

^a^Sensitivity measures the proportion of detachments that are correctly predicted as detachments.

**Figure 1:**
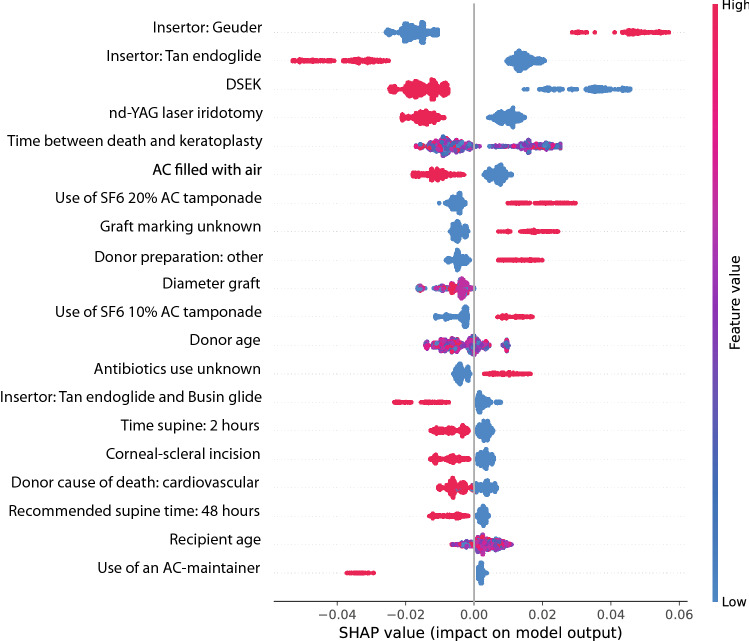
Summary of the SHAP values based on the random forest classifier model. The y-axis shows the most relevant features for predicting graft detachment, from highest to lowest. The x-axis displays the SHAP values, reflecting the effect of each variable on the model’s outcome by indicating the predicted change in the probability of a graft detachment. A negative SHAP value represents a “protective effect” (i.e., decreased risk of detachment), while a positive SHAP value represents a “risk effect” (i.e., increased risk of detachment). Each symbol in the plot represents an individual patient in the test dataset. The color gradient ranging from blue to red represents the range of values of the variables. For binary variables, only two colors are used (red for “yes” and blue for "no"); for continuous variables, the values are depicted using the entire spectrum from red to blue.

An overview of each model output is presented in Fig. 1 for the RFC model and in Supplementary Figs. S1 and S2 for the lasso and CTA models, respectively. The three models identified different sets of predictive factors, although several factors overlapped between the three models. The results of the most predictive factors identified by the three models are shown in Table 3. To simplify the analysis, factors that were categorized as either “unknown” or “unspecified” were omitted from Table 3.

**Table 3:**
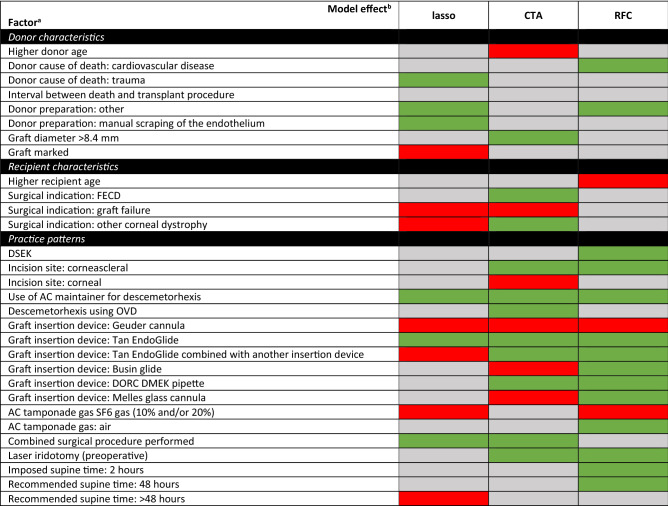
Overview of the most relevant factors and their effect identified by the three machine learning models (green: reduced risk, red: increased risk, light grey: unclear).

*AC* anterior chamber, *CTA* classification tree analysis, *DORC* Dutch Ophthalmic Research Center, *FECD* Fuchs endothelial corneal dystrophy, *LASSO* logistic regression using least absolute shrinkage and selection operator, *OVD* ocular viscoelastic device, *RFC* random forest classification, *SF6* sulfur hexafluoride.

^a^Factors with the category “unknown” or “unspecified” have been omitted.

^b^Green: reduced risk of detachment; red: increased risk of detachment; light grey: unclear (either diffuse pattern or not identified by the model).

We found that undergoing DMEK was associated with an increased risk of graft detachment in all three models. In contrast, none of the three models showed that performing a combined procedure was associated with an increased risk of postsurgical complications; however, both the LASSO and CTA models found that undergoing a combined procedure reduced the risk of graft detachment. In addition, the outcome was unclear for several factors, either because the model did not identify the factor as important or because the pattern was diffuse (see Fig. 1). Risk factors that were common to at least two of the three models included a previous graft failure, the type of insertion device, and the use of sulfur hexafluoride (SF6) gas during surgery. Protective factors that were common to at least two models included “donor preparation: other”, use of an AC maintainer during descemetorhexis, and combining surgical procedures (all combinations of procedures). It should be noted that by design, the type of insertion device is specific to the procedure (either DSEK or DMEK) being performed. In addition, it is important to note that “donor preparation method: other” is often entered as the method for preparing the graft for DMEK; based on a case-by-case assessment, we believe this is simply another way of saying “pre-cut tissue”. Similarly, “manual scraping of the endothelium” is likely another way of saying “pre-cut tissue” for DMEK.

## Discussion and conclusions

Here, we report the use of three different machine learning models to explore factors that can predict the probability of graft detachment following posterior lamellar keratoplasty. The predictive power was similar between all three models and is considered to be acceptable (ranging from 0.65 to 0.72). Our identification of predictive factors can help surgeons make evidence-based changes of their practice patterns, provide insight in the marginal contribution to surgical safety of current practices, and can help generate hypotheses for empirical clinical research regarding the origins of graft detachments. Particularly, this study can function as a data-driven protocol standardization of future prospective studies regarding posterior lamellar keratoplasties.

A major strength of this study is our access to an extensive, nationwide dataset and the inclusion of practice patterns in our analysis of a national cornea transplantation registry. The real-world data of the transplantation registry are represented with practically absent inclusion bias, owing to the obligatory and incentivized data-entry in the register. The assessment of both linear and non-linear relationships between a wide range of factors is unique and revealed the complex interactions between factors^8,11,12,27,36^. Moreover, the presented approach enables the evaluation of the numerous modulations of practice patterns in use and the relative impact of proposed key factors, particularly of value in the rapidly evolving field of surgery in which assessing these practice patterns independently in a clinical study would not be feasible.

Despite these strengths several limitations warrant discussion. None of our three models explain all of the variance in our dataset, and not all factors that can affect graft detachment are registered in our dataset (e.g., patient’s behavior and compliance, unrecorded intraoperative events). Furthermore, the register lacks comprehensive contextual information and the completeness and correctness of the data in the registry could not be validated. This lack of contextual information is exemplified by our finding of unknown and/or missing observations in the model output; the interpretation of which is ambiguous and full of assumptions. We therefore chose to not report these outputs. With respect to the practice patterns, we cannot exclude response/recall bias regarding the replies to the questionnaire. Although the surgical protocols for DSEK and DMEK overlap to a large degree, they also differ in several respects. By aggregating the two procedures into a single database, procedure-specific predictive factors might not be identified. Certain predictive factors, such as aphakia, were relatively rare and therefore lack the necessary power to appear in the model output, whilst experts agree on the added risk of this particular condition. Therefore, the effect of certain variables cannot be estimated reliably. Finally, the retrospective nature of the study should be noted. Prospective validation of the results is essential to evaluate the usefulness of the models and possible applications in clinical practice.

The effect of donor-related factors, recipient-related factors, surgery-related factors, and practice patterns on the prevalence of graft detachment is an ongoing topic of discussion. Many surgeons in the Netherlands are transitioning from DSEK to DMEK^37^, and our results indicate that DMEK is associated with an increased risk of graft detachment, consistent with previous studies^4–6^. This increased risk may be due in part to increased difficulty when handling the DMEK graft and/or the fact the DMEK graft edge is more prone to curling up, thus lifting the graft from the recipient’s stromal bed^38,39^. In addition, partial detachments are more common after DMEK, possibly increasing the rate of rebubbling of the graft compared to DSEK^40^. Alternatively, the increased risk associated with DMEK may partially be related to the surgeon’s learning curve. Indeed, from 2016 to 2018 the prevalence of graft detachment decreased more steeply for DMEK than for DSEK^4^. In our model, we attempted to correct for this learning curve by excluding the first 20 DMEK surgeries performed at each clinic, although the results in Table 1 suggest a shallower learning curve; thus, our model may have overestimated the effect of the DMEK procedure and DMEK specific factors.

Our results show a diffuse pattern of donor age, recipient age, donor cause of death, and the interval between donor death and surgery. Regarding these factors, our analyses are inconclusive. Regarding preparation techniques, our results are consistent with previous studies that found no difference between pre-cut and surgeon-cut tissues^13,15^. Graft marking was associated with an increased risk of detachment in the Lasso model only. However, in the Netherlands graft marking is infrequently practiced, clouding the full assessment of the effect of this practice. Furthermore, and consistent with previous findings, our models indicate that patients who had one or more previously failed grafts had a higher risk of detachment^36^.

We also found that several types of graft insertion devices were associated with an increased risk graft detachment; however, we consider the choice of insertion device a proxy for idiosyncratic surgeon factors too subtle to be captured in our register or questionnaire. We opted not to enter to individual surgeon or center as a model factor, as this study is not designed as an exercise in benchmarking. Notwithstanding, these expected between-surgeon differences might now be attributed to proxy parameters. The insertion tools themselves are known to increase the risk of endothelial damage, although no significant differences have been found between the various commercially available insertion devices^8,20,21^. Furthermore, we found that a graft diameter > 8.4 mm may be associated with a reduced risk of graft detachment. Several groups previously hypothesized that a larger graft may overlap with the retained Descemet membrane in the recipient, thus inhibiting graft attachment^22,23^. However, no effect of graft size compared to the descemetorhexis size was found. Both DMEK and DSEK are increasingly combined with other procedures such as cataract surgery. In none of our models a combination of procedures was associated with an increased risk risk of graft detachment. Combining these results with previous studies we can conclude that combining surgical procedures does not increase the risk of graft detachment^12,25,26^. Finally, two of the three models in our study found that pre-operative laser peripheral iridotomy was more protective than surgical peripheral iridectomy, although none of the models found that surgical peripheral iridectomy substantially increased the risk of detachment. This difference between laser iridotomy and surgical iridectomy may be due to the increased risk of intraoperative fibrin formation during surgical iridectomy^41^.

Interestingly, we found that using air as the tamponade agent was not associated with an increased risk of graft detachment, while using SF6 gas appeared to increase the risk of graft detachment. This finding is in contrast with previous studies suggesting that the use of SF6 gas may reduce the risk of graft detachment^32,42,43^. This discrepancy may be explained in part by the recent transition of surgeons to using SF6 gas together with the concomitant transition to performing DMEK (with a subsequent increased risk of detachment in their learning curve). Nevertheless, we believe that the previously reported putative benefits associated with using SF6 gas might have been overestimated relative to all other factors and is exemplified by continued reports of relatively high rates of graft detachment^5,6,12,40,44,45^. After posterior lamellar keratoplasties, patients are instructed to remain in the supine position in order to maximize the beneficial effects of AC tamponade, and the length of time in this position can affect the risk of graft detachment. The results of our study indicate that strictly imposing a supine duration of at least 2 h reduced the risk of graft detachment. Similar results were also found if the patients were instructed to remain in the supine position for 48 h following surgery, consistent with the routine practice of most surgeons^9,28^.

Lastly, several previously suggested risk and protective factors were not identified by our models. For example, we found no effect of increasing intraocular pressure above physiological limits for a certain time, consistent with previous studies suggesting that overpressuring of the eye after graft insertion has only a limited protective effect^30,46,47^. Similarly, we found no increased risk of complications either during or following surgery; however, this apparent lack of effect may have been due to the relatively low incidence of these events.

In conclusion, we applied a supervised machine learning approach to a nationwide dataset and identified the most relevant factors for predicting graft detachment following posterior lamellar keratoplasties. Our analysis revealed that performing a DMEK procedure, the use of SF6 gas, and previous graft failure increased the risk of detachment, whereas performing a DSEK procedure, preoperative laser iridotomy, larger graft size, remaining strictly supine for at least 2 h, and a recommendation for staying in the supine position for 48 h reduced the risk of detachment. In contrast, performing a combined procedures and the use of pre-cut tissue had no effect on the risk of graft detachment, neither did overpressuring of the eye after graft-insertion. These results can help surgeons improve their practice patterns and can help researchers formulate new, testable hypotheses. Future studies should focus on improving the performance of machine learning approaches by including more detailed, contextual information. Importantly, these models’ “in silico” predictions should be tested in clinical practice.

## Methods

### Data collection

The data used in this study were acquired from the NOTR, which is hosted by the Netherlands Transplant Foundation (NTS). We included all DSEK and DMEK procedures registered in the NOTR between January 1, 2015, and December 31, 2018, including 12 months of follow-up data. The Netherlands Institute for innovative ocular surgery did not participate in the nationwide registry at the time of the data collection (2015–2018) and their data is therefore not included in this analysis. Two cornea banks (Amnitrans EyeBank, Rotterdam and the ETB-bislife, Beverwijk) supplied all of the corneal grafts assessed in this study. The NOTR steering committee provided Institutional Review Board (IRB) approval for the extraction and analysis of data in this study. All patients provided informed consent to be included in the registry for research purposes. No identifying information of donors or patients was available to the researchers and all data were anonymized prior to delivery to the researchers. No donor tissue was were procured from prisoners. In accordance with IRB approval, the data were not stratified at the individual surgeon, center, donor or patient level. The study was conducted in accordance with the principles of the Declaration of Helsinki and Dutch legislation. The NOTR data were restructured and made accessible for machine learning analysis.

### Registry data processing

All available data in the registry regarding the donor, graft, recipient, and practice patterns was collected. The donor’s information included sex, age at the time of death, cause of death, endothelial cell count, interval between death and explantation of the eye, interval between explantation of the eye and preservation of the eye, and interval between death and the transplant procedure. The donor’s cause of death was classified into the following five categories: neoplasms/cancer, diseases of the respiratory system, trauma, diseases of the cardiovascular system, and other causes of death. The recipient’s information included sex, age at the time of surgery, indication for corneal transplantation surgery, and preoperative lens status. The indication for transplant surgery was classified into the following five categories: Fuchs corneal endothelial dystrophy (FECD), pseudophakic bullous keratopathy (PKB), graft failure, other corneal dystrophies, and other indications. Information regarding the surgical procedure included the surgeon’s position (staff surgeon or surgical fellow), date of surgery, instruments used for donor preparation, instruments used for graft insertion, diameter of the donor graft, diameter of the descemetorhexis, whether it was a combined surgical procedure, and surgical complications. Combined surgical procedures were recoded into the following five groups: peripheral surgical iridectomy, cataract surgery, posterior intraocular lens insertion without cataract extraction, anterior vitrectomy, and other combined surgical procedures or unspecified. Postoperative events recorded in the NOTR were classified as surgery-related (e.g., rebubbling, graft failure, immunological reaction, iatrogenic glaucoma, and/or cystoid macula edema) and not surgery-related (e.g., intravitreal injections, posterior segment surgery after primary transplant, and/or extra-ocular events). The recoding of the variables resulted in a set of 91 predictor variables.

### Practice pattern questionnaire

We used a questionnaire to determine the practice patterns used by the transplantation centers that contributed their data to the registry, including the center-specific practice patterns (e.g., method of iridectomy, instruments used during surgery, AC tamponade, and supine time) and any protocol changes that may have occurred within the data collection period. All practice patterns questionnaires were collected by the NTS and anonymized before delivery to the researchers.

The center-specific practice patterns were connected to the respective patients in the registry, and protocol changes that occurred in the period between January 2015 and December 2018 were taken into account. To reduce the potential effects of a surgical learning curve, the first 20 DMEK surgeries performed at each center were removed from the dataset^4^.

### Machine learning approach

The primary outcome measure of this study was postoperative graft detachment, which was defined as the occurrence of an intervention to re-adhere the graft (i.e., the incidence of rebubbling) reported in the NOTR. The dataset was divided into a training set and a test set, compromised of 70% and 30% of the dataset, respectively. The training set was used to develop a suitable model based on the predictive variables identified, and the test set was used to validate the model. The following three machine learning models were built: a L1 regularized logistic regression using least absolute shrinkage and selection operator (lasso) model, a classification tree algorithm (CTA), and random forest classification (RFC).

These three models have been chosen for the following reasons. Given the 91 predictors, a simple logistic regression analysis with outcome graft detachment is computationally difficult or even impossible. The lasso model is a special form of logistic regression in which the estimated regression coefficients are shrunken towards zero relative to the least squares estimates. As a result, some coefficients will be exactly zero, which leads to the selection of a subset of most predictive predictors for graft detachment. However, the Lasso will only be able to detect linear relations of the predictors with the outcome detachment. To detect non-linear relationships and higher-order relationships among the explanatory variables, we used CTA and RFC. The CTA partitioned the training dataset based on outcome (i.e., graft detachment/no graft detachment) using a series of successive splits (i.e., nodes)^48^. For each split, the explanatory variable that best partitioned the records was chosen based on accuracy, until the set could not be split further. To reduce over-fitting, pruning was performed using fivefold cross-validation, thus removing nodes that did not improve the accuracy of the tree. In the final tree, each leaf node was assigned the class with the highest frequency among its records, and each record reaching the node was predicted as being in that class. Although classification trees are very useful to detect higher-order relations and non-linear relations, they are not very robust, meaning that small changes in the data can result in large changes in the final estimated tree. RFC leads to a more robust classification model by building a large number of classification trees and splitting the data using a random sample of the entire set of explanatory variables to serve as split candidates. The resulting trees were combined by taking a majority vote, and the overall prediction was the most frequently occurring class among all predictions. By forcing each split to consider only a subset of variables, the RFC analysis overcomes the potential problem of one or more strong predictors dominating the solution, thus rendering the average of the trees less variable and therefore more reliable. In this study 1000 trees were used to build the RFC model.

The relatively low rate of graft detachment in the dataset resulted in a large imbalance between the two outcome categories (i.e., detachment versus no detachment) and can therefore affect the statistical model estimation and evaluation. To solve this imbalance, we performed random oversampling of examples (ROSE) for the lasso and the CTA^49^. The RFC model was trained in combination with weights to balance the outcome classes. No resampling techniques were used for the test set.

### Statistical analysis

We summarized all quantitative and qualitative variables, including the donor characteristics, recipient characteristics, procedure characteristics, postoperative events, and practice patterns. The prevalence of graft detachment was determined separately for all procedures involving DSEK and all procedures involving DMEK independently. All statistical analyses were performed using the R statistical software package version 4.0.5 (Comprehensive R Archive Network, Vienna, Austria), except for the RFC which was performed using in Python version 3.8 and the scikit-learn package version 0.24.1 (Python Software Foundation. Python Language Reference, version 2.7).

The machine learning models were evaluated using the test set. The predicted outcome was compared with the observed outcome reported in the NOTR (i.e., the ground truth) by calculating the sensitivity, specificity, and the area under the curve (AUC). As different machine learning methods were used it was expected the models would diverge to some extend and a qualitative analysis of the model outcomes was performed. The overlap between models was used to identify factors associated with an increased or decreased risk.

## Supplementary Information

Supplementary Information 1.

Supplementary Information 2.

Supplementary Information 3.

Supplementary Information 4.

Supplementary Information 5.

Supplementary Information 6.

Supplementary Information 7.

## Data Availability

The data that support the findings of this study are available from the Netherlands Organ Transplant Registry but restrictions apply to the availability of these data, which were used under license for the current study, and are not publicly available.
