# Supplementary material for: A machine learning approach to explore predictors of graft detachment following posterior lamellar keratoplasty: a nationwide registry study
Source: Sci Rep. 2022 Oct 21;12:17705. doi: 10.1038/s41598-022-22223-y (PMC9586999; doi:10.1038/s41598-022-22223-y)
Supplement: Supplementary file 1 — Supplementary Information 1. [file 41598_2022_22223_MOESM1_ESM.docx]

| **Supplementary Table S1: Practice patterns for the 11 cornea centers that performed DSEK in the study period.** | | | | | | | | | | |  |  |
| --- | --- | --- | --- | --- | --- | --- | --- | --- | --- | --- | --- | --- |
|  |  | **Cornea center** | | | | | | | | | | |
|  |  | **1** | **2** | **3** | **4** | **5** | **6** | **8** | **9** | **11** | **14** | **15** |
| **Iridectomy method** | YAG | ● | ● | ● | ● |  |  | ● | ● | ● | ● | ● |
|  | Mantoux needle |  |  |  |  | ● | ● | ● |  |  | ● |  |
|  | Segment |  |  |  |  |  |  |  | ● | ● |  |  |
| **Descemetorhexis** |  |  |  |  |  |  |  |  |  |  |  |  |
| General radius | ≤8.0 mm | ● |  | ● |  |  |  |  |  |  |  | ● |
|  | 8.5 mm |  |  |  |  | ● |  |  | ● |  |  |  |
|  | 9.0 mm |  |  |  | ● |  | ● | ● |  | ● | ● |  |
|  | ≥9.5 mm |  | ● |  |  |  |  |  |  |  |  |  |
| Use of dye |  | No | No | No | No | No | Yes | No | Yes | No | No | No |
| Anterior chamber filling | Air | ● |  | ● | ● |  | ● | ● |  | ● |  |  |
|  | BSS |  |  |  |  |  |  |  | ● |  |  |  |
|  | Active AC infusion with air |  | ● |  |  | ● |  |  |  |  |  | ● |
|  | Viscoelastic device |  |  |  |  |  |  |  |  |  | ● |  |
| Additional interventions | None |  |  | ● |  | ● |  |  | ● |  |  |  |
|  | Exchange air with fluid | ● | ● |  |  |  |  |  |  |  |  |  |
|  | Fill AC with air |  |  |  | ● |  |  | ● |  | ● |  | ● |
|  | Recolor with a dye |  |  |  |  |  | ● |  |  |  |  |  |
|  | Polish with I/A |  |  |  |  |  |  | ● |  |  | ● |  |
| Graft insertor | Tan EndoGlide | ● | ● | ● | ● |  | ● | ● |  |  | ● | ● |
|  | Busin glide |  |  | ● |  | ● |  | ● | ● | ● | ● |  |
|  | Maculoso injector |  |  |  |  |  | ● |  |  |  |  |  |
| Incision site | Sclera | ● | ● |  |  |  |  |  | ● | ● |  | ● |
|  | Cornea-sclera |  |  |  | ● | ● | ● | ● |  |  |  |  |
|  | Cornea |  |  | ● |  |  |  | ● |  |  | ● |  |
| Incision size (mm) |  |  |  | 3.5 | 5.0 |  | 4.5 |  | 4.0 | 4.5 | 4.5 | 4.5 |
| Overpressure time (minutes) | 0 | ● | ● |  |  |  | ● |  | ● |  |  |  |
|  | <10 |  |  |  |  |  |  |  |  |  | ● |  |
|  | ≥10 |  |  | ● | ● | ● |  | ● |  | ● |  | ● |
| Intracameral antibiotics |  | No | No | Yes | No | missing | No | No | missing | Yes | Yes | No |
| **Postoperative AC tamponade** |  |  |  |  |  |  |  |  |  |  |  |  |
| Filling | Air | ● | ● | ● |  | ● |  | ● | ● | ● |  |  |
|  | SF6 10% |  |  |  | ● |  |  |  |  |  |  | ● |
|  | SF6 20% |  |  |  |  |  | ● |  |  |  | ● |  |
| Time | Until resorption | ● | ● | ● | ● | ● | ● | ● | ● | ● | ● | ● |
| Strict time supine after surgery | 1 hour |  |  |  |  | ● |  |  |  |  |  |  |
|  | 2 hours |  |  | ● | ● |  | ● |  |  |  | ● |  |
|  | 3 hours |  |  |  |  |  |  | ● |  |  |  | ● |
|  | 4 hours |  |  |  |  |  |  |  | ● |  |  |  |
|  | 24 hours | ● |  |  |  |  |  |  |  |  |  |  |
|  | 48 hours |  |  |  |  |  |  |  |  | ● |  |  |
|  | Until resorption |  | ● |  |  |  |  |  |  |  |  |  |
| Recommended time supine after discharge | <24 hours |  |  | ● |  | ● |  |  |  |  |  |  |
|  | 24 hours |  |  |  |  |  |  |  | ● |  | ● |  |
|  | 48 hours |  |  |  | ● |  | ● |  |  | ● |  |  |
|  | >48 hours |  | ● |  |  |  |  |  |  |  |  | ● |

NA, not applicable.
