# Supplementary material for: A machine learning approach to explore predictors of graft detachment following posterior lamellar keratoplasty: a nationwide registry study
Source: Sci Rep. 2022 Oct 21;12:17705. doi: 10.1038/s41598-022-22223-y (PMC9586999; doi:10.1038/s41598-022-22223-y)
Supplement: Supplementary file 3 — Supplementary Information 3. [file 41598_2022_22223_MOESM3_ESM.docx]

| **Supplementary Table S3. Donor characteristics.** | | | |
| --- | --- | --- | --- |
|  | **All (n=3647)** | **DSEK (n=2651)** | **DMEK (n=996)** |
|  | N (%) | N (%) | N (%) |
| **Demographics** |  |  |  |
| Gender male | 2238 (61.4) | 1607 (60.6) | 631 (63.4) |
| Age in years, mean ± SD | 70 ± 9 | 69 ± 9 | 72 ± 8 |
| **Donor cause of death** |  |  |  |
| Neoplasms/cancer | 320 (8.8) | 246 (9.3) | 74 (7.4) |
| Diseases of the circulatory system | 1945 (53.3) | 1476 (55.7) | 469 (47.1) |
| Diseases of the respiratory system | 742 (20.3) | 478 (18) | 264 (26.5) |
| Trauma | 584 (16) | 410 (15.5) | 174 (17.5) |
| Other | 56 (1.5) | 41 (1.5) | 15 (1.5) |
| **Donor preparation** |  |  |  |
| Manual scraping of the endothelium | 792 (21.7) | 732 (27.6) | 60 (6) |
| Manual lamellar dissection | 1131 (31) | 1101 (41.5) | 30 (3) |
| Pre-cut | 1496 (41) | 814 (30.7) | 682 (68.5) |
| Other | 228 (6.3) | 4 (0.2) | 224 (22.5) |
| **Graft preservation medium** |  |  |  |
| CorneaMax | 1185 (32.5) | 810 (30.6) | 375 (37.7) |
| Culture medium | 2462 (67.5) | 1841 (69.4) | 621 (62.3) |
| **Graft preservation time** |  |  |  |
| Interval between death and explantation of the donor tissue in minutes, mean ± SD | 645 ± 344 | 652 ± 344 | 627 ± 342 |
| Interval between explantation and preservation of the donor tissue in minutes, mean ± SD | 807 ± 447 | 794 ± 454 | 843 ± 424 |
| Interval between death of the donor and corneal transplantation in days, mean ± SD | 19 ± 5 | 19 ± 5 | 20 ± 5 |
