# Supplementary material for: A machine learning approach to explore predictors of graft detachment following posterior lamellar keratoplasty: a nationwide registry study
Source: Sci Rep. 2022 Oct 21;12:17705. doi: 10.1038/s41598-022-22223-y (PMC9586999; doi:10.1038/s41598-022-22223-y)
Supplement: Supplementary file 4 — Supplementary Information 4. [file 41598_2022_22223_MOESM4_ESM.docx]

| **Supplementary Table S4. Recipient characteristics.** | | | |
| --- | --- | --- | --- |
|  | **All (n=3647)** | **DSEK (n=2651)** | **DMEK (n=996)** |
|  | N (%) | N (%) | N (%) |
| **Recipient age in years, mean ± SD** | 73 ± 11 | 74 ± 11 | 73 ± 9 |
| **Surgery indication** |  |  |  |
| Fuchs endothelial dystrophy | 2772 (76) | 1866 (70.4) | 906 (91) |
| Pseudophakic bullous keratopathy | 362 (9.9) | 330 (12.4) | 32 (3.2) |
| Graft failure | 250 (6.9) | 231 (8.7) | 19 (1.9) |
| Other corneal dystrophies | 82 (2.2) | 66 (2.5) | 16 (1.6) |
| Other | 148 (4.1) | 126 (4.8) | 22 (2.2) |
| **Lens status (pre-surgery)** |  |  |  |
| Phakic | 539 (14.8) | 437 (16.5) | 102 (10.2) |
| Anterior chamber IOL | 136 (3.7) | 115 (4.3) | 21 (2.1) |
| Posterior chamber IOL | 2273 (62.3) | 1574 (59.4) | 669 (70.2) |
| IOL not specified | 18 (0.5) | 12 (0.5) | 6 (0.6) |
| Aphakic | 24 (0.7) | 23 (0.9) | 1 (0.1) |
| Unknown | 657 (18) | 490 (18.5) | 167 (16.8) |
| **Previous corneal transplant in the same eye** |  |  |  |
| No previous transplant | 3240 (88.8) | 2285 (86.2) | 995 (95.9) |
| One previous transplant | 354 (9.7) | 317 (12) | 37 (3.7) |
| Two previous transplants | 48 (1.3) | 45 (1.7) | 3 (0.3) |
| Three previous transplants | 5 (0.1) | 4 (0.2) | 1 (0.1) |
| IOL, intraocular lens. | | | |
