# Supplementary material for: A machine learning approach to explore predictors of graft detachment following posterior lamellar keratoplasty: a nationwide registry study
Source: Sci Rep. 2022 Oct 21;12:17705. doi: 10.1038/s41598-022-22223-y (PMC9586999; doi:10.1038/s41598-022-22223-y)
Supplement: Supplementary file 5 — Supplementary Information 5. [file 41598_2022_22223_MOESM5_ESM.docx]

| **Supplementary Table S5. Surgical details** | | | |
| --- | --- | --- | --- |
|  | **All (n=3647)** | **DSEK (n=2651)** | **DMEK (n=996)** |
|  | N (%) | N (%) | N (%) |
| **Operating surgeon** |  |  |  |
| Fellow | 172 (4.7) | 162 (6.1) | 10 (1) |
| Staff | 3475 (95.3) | 2489 (93.9) | 986 (99) |
| **Graft size compared to descemetorhexis size** |  |  |  |
| Undersized | 953 (26.1) | 548 (20.7) | 405 (40.7) |
| Same size | 1439 (39.5) | 1006 (37.9) | 433 (43.5) |
| Oversized | 1255 (34.4) | 1097 (41.4) | 158 (15.9) |
| **Combined surgical procedure** |  |  |  |
| None | 2615 (71.7) | 1843 (69.5) | 772 (77.5) |
| Peripheral iridectomy | 444 (12.2) | 302 (11.4) | 142 (14.3) |
| Cataract surgery | 313 (8.6) | 273 (10.3) | 40 (4) |
| Peripheral iridectomy and cataract surgery | 132 (3.6) | 100 (3.8) | 32 (3.2) |
| Lens implant without cataract extraction | 71 (1.9) | 64 (2.4) | 7 (0.7) |
| Anterior vitrectomy | 7 (0.2) | 6 (0.2) | 1 (0.1) |
| Other | 65 (1.8) | 63 (2.4) | 2 (0.2) |
| **Surgical complications** |  |  |  |
| Incidence of complications | 148 (4.1) | 88 (3.3) | 60 (6) |
| Endothelial damage | 28 (0.8) | 13 (0.5) | 15 (1.5) |
| Graft unfolding problems | 24 (0.7) | 5 (0.2) | 19 (1.9) |
| Rupture of graft tissue and other preparation problems | 16 (0.4) | 8 (0.3) | 8 (0.8) |
| Iris prolapse | 16 (0.4) | 16 (0.6) | NA |
| Anterior chamber hemorrhage | 11 (0.3) | 6 (0.2) | 5 (0.5) |
| Graft prolapse or expulsion | 10 (0.3) | 4 (0.2) | 6 (0.6) |
| Vitreous loss | 8 (0.2) | 6 (0.2) | 2 (0.2) |
| Graft dislocation or adherence problems | 5 (0.1) | 5 (0.2) | NA |
| Failed/aborted procedure | 5 (0.1) | 4 (0.2) | 1 (0.1) |
| Decentered trephination | 4 (0.1) | 3 (0.1) | 1 (0.1) |
| Rupture of the posterior lens capsule | 3 (0.1) | 3 (0.1) | NA |
| Lens touch (unintended) | 1 (0.03) | NA | 1 (0.1) |
| Zonulolysis | 1 (>.03) | 1 (0.04) | NA |
| Other | 16 (0.4) | 14 (0.5) | 2 (0.2) |

NA, not applicable.
